# Voluntary activity reverses spermidine-induced myocardial fibrosis and lipid accumulation in the obese male mouse

**DOI:** 10.1007/s00418-020-01926-1

**Published:** 2020-10-27

**Authors:** Christian Mühlfeld, Clara Pfeiffer, Vanessa Schneider, Melanie Bornemann, Julia Schipke

**Affiliations:** 1 Institute of Functional and Applied Anatomy, Hannover Medical School, Carl-Neuberg-Str. 1, 30625 Hannover, Germany; 2 Biomedical Research in Endstage and Obstructive Lung Disease Hannover (BREATH), German Center for Lung Research (DZL), Hannover, Germany

**Keywords:** Obesity, Spermidine, Voluntary activity, Hypertrophy, Stereology, Myocardium, Heart

## Abstract

Obesity due to high calorie intake induces cardiac hypertrophy and dysfunction, thus contributing to cardiovascular morbidity and mortality. Recent studies in aging suggest that oral supplementation with the natural polyamine spermidine has a cardioprotective effect. Here, the hypothesis was tested that spermidine or voluntary activity alone or in combination protect the heart from adverse effects induced by obesity. Therefore, C57Bl/6 mice (*n* = 8–10 per group) were subjected to control or high fat diet (HFD) and were left untreated, or either received spermidine via drinking water or were voluntarily active or both. After 30 weeks, the mice were killed and the left ventricle of the hearts was processed for light and electron microscopy. Design-based stereology was used to estimate parameters of hypertrophy, fibrosis, and lipid accumulation. HFD induced cardiac hypertrophy as demonstrated by higher volumes of the left ventricle, cardiomyocytes, interstitium, myofibrils and cardiomyocyte mitochondria. These changes were not influenced by spermidine or voluntary activity. HFD also induced myocardial fibrosis and accumulation of lipid droplets within cardiomyocytes. These HFD effects were enhanced in spermidine treated animals but not in voluntarily active mice. This was even the case in voluntarily active mice that received spermidine. In conclusion, the data confirm the induction of left ventricular hypertrophy by high-fat diet and suggest that—under high fat diet—spermidine enhances cardiomyocyte lipid accumulation and interstitial fibrosis which is counteracted by voluntary activity.

## Introduction

Modern life-style is increasingly accompanied by a misrelationship between calorie intake and expenditure. The resulting obesity is frequently associated with chronic diseases, such as diabetes type II, metabolic syndrome, or cardiomyopathy (Ahima 2011; Pegklidou et al. 2010), and thus with a higher mortality rate (Arnlov et al. 2010). In the heart, obesity is linked to left ventricular hypertrophy, accumulation of fat, apoptosis of cardiomyocytes, fibrosis, and systolic and diastolic dysfunction (Abel et al. 2008; Gruber et al. 2012; Harmancey et al. 2008; Schipke et al. 2014). The adverse effects of the higher lipid burden are summarized by the term lipotoxicity (Unger et al. 2010; Sletten et al. 2018). Naturally, the combination of reduced calorie intake (by eating less and differently) and increased calorie expenditure (by more physical activity) would be the most successful approach against the development and the consequences of obesity, yet it usually proves to be difficult. Therefore, pharmaceuticals or nutritional supplements supporting weight loss or the beneficial effects of physical activity would help fight the growing health burden of obesity.

A new therapeutic approach could be the oral supplementation with polyamines, such as putrescine, spermine or spermidine. Polyamines are synthesized in nearly all cells of the mammalian (including human) body and some foods are particularly rich in polyamines like soy beans or wheat germ (Soda et al. 2009a). Polyamines are involved in various important cellular processes like apoptosis (Bjelakovic et al. 2010), cell division (Ray et al. 1999) and autophagy (Madeo et al. 2010). This has evoked studies addressing the potential of polyamines as anti-aging drugs. Spermidine was shown to increase the life time of yeast and human cells as well as organisms like drosophila and mice (Eisenberg et al. 2009; Soda et al. 2009b). In mice, the life-prolonging effect of spermidine could at least partly be attributed to a cardioprotective effect (Eisenberg et al. 2016).

Evidence suggests that spermidine might also exert beneficial effects on obesity itself or its cardiac consequences. An accelerated polyamine flux in transgenic mice leads to lower acetyl- and malonyl-CoA levels, increased glucose- and palmitate-oxidation and higher basal metabolic rates ultimately resulting in reduced white adipose tissue, improved glucose tolerance and a distinct lean phenotype (Jell et al. 2007; Pirinen et al. 2007). In contrast, abrogation of the polyamine catabolism exacerbates the accumulation of body fat demonstrating the close association between polyamine metabolism and body fat and energy homeostasis (Jell et al. 2007). Interestingly, mice offered a polyamine-rich chow consumed significantly more food than controls but had similar body weights and seemed to be more active (Soda et al. 2009b). In addition, the combination of spermidine and voluntary exercise reduced the body weight of obese mice induced by high-sucrose diet whereas spermidine alone had no effect (Schipke et al. 2019). Autophagy as a cellular degradation pathway plays a critical role for lipid droplet degradation, and an abnormal intracellular lipid increase impairs the autophagic clearance. Studies suggest that autophagy is also impaired in the obese heart contributing to obesity-induced cardiac dysfunction, thus spermidine as autophagy inducing agent might affect the cardiac lipid accumulation in obesity. Based on the previous considerations, we hypothesized that spermidine alone or the combination with voluntary exercise alleviates the cardiac changes induced by high-fat diet in male mice. We used a well-established obesity model of long-term high-fat diet in the mouse (Hollenbach et al. 2019; Schipke et al. 2019; Ahrendt et al. 2020) and analyzed the left ventricular myocardium by light and electron microscopy with respect to structural parameters that had previously been shown to be altered in obesity and/or beneficially changed by spermidine in aging (Gruber et al. 2012; Eisenberg et al. 2016).

## Material and methods

### Mice

This study was conducted in accordance with the German animal protection law and with the European Directive 2010/63/EU. All animal experiments were approved and permitted by the Lower Saxony State Office for Consumer Protection and Food Safety (LAVES; file number: 13/1244). Male C57BL/6 N mice were purchased at the age of 5 weeks from Charles River (Sulzfeld, Germany) and—after one week of acclimatization—were randomly assigned to one of the following eight experimental groups: control or high-fat diet (CD, *n* = 10 or HFD, *n* = 9), control diet plus spermidine (CD-S, *n* = 8 or HFD-S, *n* = 8), control or high-fat diet plus voluntary activity (CD-A, *n* = 10 or HFD-A, *n* = 9), control or high-fat diet plus spermidine and voluntary activity (CD-A-S, *n* = 9 or HFD-A-S, *n* = 9). The experiments were started with an equal number of mice per group (*n* = 10). Due to various reasons some animals had to be excluded from the analyses (Table 1) which explains the different number of mice per group. Control groups received a control diet consisting of 70 kcal% carbon hydrates (7 g% sucrose), 20 kcal% proteins and 10 kcal% fat for 30 weeks (D12450J, Research Diets, USA). High-fat diet groups received a diet consisting of 20% carbon hydrates (7% sucrose), 20% proteins and 60% fat for 30 weeks (D12492, Research Diets, USA). Spermidine groups with or without exercise were administered 3 mmol/l spermidine (Sigma-Aldrich) via the drinking water. The spermidine-supplemented drinking water was covered from light and renewed every third or fourth day. Activity groups with or without spermidine supplementation had access to a running wheel throughout the experimental period (Mini Mitter, Starr Life Science Group, USA). All animals were kept in single cages at a 12 h:12 h day and night rhythm and had ad libitum access to food and drinking water. At the end of the experiments, the mice were weighed and subsequently anesthetized with ketamine (100 mg/kg b.w.) and xylazine (5 mg/kg b.w.) and killed by exsanguination. The animals are part of a larger cohort and body weight data were previously published in Ahrendt et al. (2020), Hollenbach et al. (2019) and Schipke et al. (2019).

**Table 1:** Number of mice per group and reasons for exclusion

Group	*n*	Exclusion reasons
CD	10	
CD-S	8	1 during experiment: spontaneous weight loss beginning in week 18, aggravating general condition, killed in week 20
		1 after finals: tumors at liver and in epididymal fat pads, enlarged kidneys and pancreas
CD-A	10	
CD-A-S	9	1 during experiment: stroke indication in week 27, killed
HFD	9	1 after finals: fluid accumulation in stomach and intestine, enlarged spleen
HFD-S	8	1 after finals: skin lesion at the side, enlarged spleen and heart
		1 after finals: skin lesion and scar tissue at the side, enlarged spleen, smaller fat depots
HFD-A	9	1 during experiment: killed because of poor general condition caused by tumor in week 22
HFD-A-S	9	1 during experiment: progressive weight loss beginning in week 20, killed in week 23

### Sample preparation

Hearts were fixed in toto by immersion in 4% paraformaldehyde in 0.2 M Hepes buffer (pH 7.4) at 4 °C. After storage for at least 24 h at 4 °C, the left ventricle was isolated and weighed. The weight of the left ventricle was divided by the density of muscle tissue, 1.06 g/cm^3^ (Mendez and Keys 1961), to obtain its volume. Then samples for light and electron microscopy were taken according to a systematic uniform random sampling scheme as described previously (Eisele et al. 2008). In brief, the left ventricle was cut twice longitudinally and three times transversally. Starting randomly with the first or second sample at the base of the ventricle, every other sample was assigned to light or electron microscopy. Samples for light microscopy were kept in the same fixative until further processing and paraffin embedding according to a standard protocol. Samples for electron microscopy were postfixed in 1.5% paraformaldehyde and 1.5% glutaraldehyde in 0.15 M Hepes buffer (pH 7.4) before further processing and epoxy resin (Agar Scientific, Essex, UK) embedding according to a standard protocol. Briefly, the samples were subsequently postfixed in 1% osmium tetroxide in 0.1 M sodium cacodylate (pH 7.4), stained en bloc with half-saturated uranyl acetate in water and dehydrated in an ascending acetone series before final embedding in epoxy resin. During the final embedding step the samples were dropped randomly into the embedding mold and left in that orientation to avoid bias from anisotropy (Stringer et al. 1982). From the paraffin blocks, 5 µm thick sections were cut, mounted on glass slides and stained with picrosirius red as described previously (Schipke et al. 2017). From three epoxy resin blocks, semi- and ultrathin sections were cut, mounted on glass or nickel mesh grids, respectively, and stained with toluidine blue or uranyl acetate and lead citrate, respectively.

### Stereology

All stereological procedures followed the guidelines and protocols described in Mühlfeld et al. (2010). At light microscopic level (100 × objective lens magnification), the volume fractions of cardiomyocytes and interstitium were estimated using a point grid superimposed on each field of view obtained by systematic uniform random sampling. For this part of the analysis a Leica DM 6000B light microscope (Leica, Wetzlar, Germany) equipped with a digital camera and attached to a computer with the newCAST software (Visiopharm, Horsholm, Denmark) was used. The sampling fraction was set to yield a number of approximately 30–60 fields of view and the final screen magnification was 2700×. At the electron microscopic level (8900 × primary magnification), the volume fractions of myofibrils, mitochondria, nuclei and sarcoplasm as well as the surface density of mitochondria were estimated using a grid consisting of eight line segments where the line end points served as point grid, i. e. 16 points. The grid was superimposed on each field of view obtained by systematic uniform random sampling and points hitting structures of interest and intersections of line segments with the outer mitochondrial membrane were counted. On the same fields of view a fine point grid consisting of 256 points was utilized to estimate the volume density of intracellular lipid droplets. Approximately, 30 electron micrographs were taken from each section, thus adding up to 90 fields of view per animal.

Additionally, a total of 60 images obtained from three samples per animal were taken by electron microscopy at a primary magnification of 11,000 × for analysis of the capillary endothelial volume and surface area. A grid consisting of 18 line segments was superimposed on the images and line end points hitting capillary endothelium, line end points hitting the reference volume and intersections of line segments with the luminal endothelial surface were counted. Images for analysis were taken with a Morgagni transmission electron microscope (Thermo Fisher Scientific, FEI, Frankfurt, Germany) and investigated using the stepanizer stereology tool (Tschanz et al. 2011).

Volume fractions (*Vv*(str/ref)) were calculated by dividing the sum of points hitting a structure of interest (*P*(str)) by the sum of points hitting the reference volume (*P*(ref)): *Vv*(str/ref) = *P*(str)/*P*(ref). The surface density of mitochondria (*Sv*(mito/cm)) was calculated based on the relationship between intersections of the line segments with the outer membrane of mitochondria (*I*(mi)) and the total length of test line hitting the reference volume, here cardiomyocytes: *Sv*(mito/cm) = 2*I/(*L*(*p*)*P(cm)), where *L*(*p*) is the line length associated with one end point of the line segment, here 1.35 µm, and *P*(cm) is the sum of line end points hitting cardiomyocytes. The arithmetic mean thickness of endothelial cells was calculated by *τ*(endo) = (*L*(*p*)*P(endo))/(2*I), where *L*(*p*) is the test line length associated with each endpoint, here 0.905 µm, *P*(endo) is the number of points hitting endothelium and *I* is the number of intersections of the test lines with the luminal surface of the endothelium (Mühlfeld and Ochs 2013).

At all levels of estimation, the point or test systems were designed to make sure that each structure receives at least 100–200 counts per animal (Gundersen and Osterby 1981, Ochs and Mühlfeld 2013). All volume and surface densities were multiplied with the reference volume to obtain total values within the left ventricle. For the calculation of cardiomyocyte and interstitial volume, the reference volume was the whole left ventricle. The volume densities of subcellular compartments as well as the surface density of mitochondria estimated by electron microscopy were related to the reference volume of cardiomyocytes estimated by light microscopy before. The reference volume of cardiomyocytes consisted of all structures belonging to the cells. The capillary estimations were related to the whole left ventricle as the reference volume.

In addition, volume and surface area of the mitochondria were used to calculate the volume-to-surface ratio of mitochondria, a parameter frequently used to evaluate the size of the mitochondria (Schmiedl et al. 1990; Mühlfeld et al. 2005). The investigators (VS, CP, MB) were blinded towards the groups the analyzed samples belonged to.

### Collagen quantification

Collagen was quantified as described previously (Schipke et al. 2017) by semiautomatic planimetry on paraffin sections stained by picrosirius red. In short, sections were digitalized at an objective lens magnification of 40 × using a slide scanner (Zeiss Axio Scan Z1, Zeiss, Oberkochen, Germany). Using the VIS software (Visiopharm, Horsholm, Denmark), the fraction of the section occupied by collagen fibers was quantified automatically based on color thresholds which had previously been adapted to avoid similarly colored structures being included in the analysis.

### Statistics

Data were analyzed by three way ANOVA followed by Tukey test for pairwise multiple comparisons using Sigma Plot (SYSTAT Software Inc., Erkrath, Germany). Linear regression analysis and Pearson correlation analysis was performed with GraphPad Prism (GraphPad Software, San Diego, CA, USA). *P* values < 0.05 were considered significant. Significant differences were indicated as explained in figure legends.

## Results

**Fig. 1:**
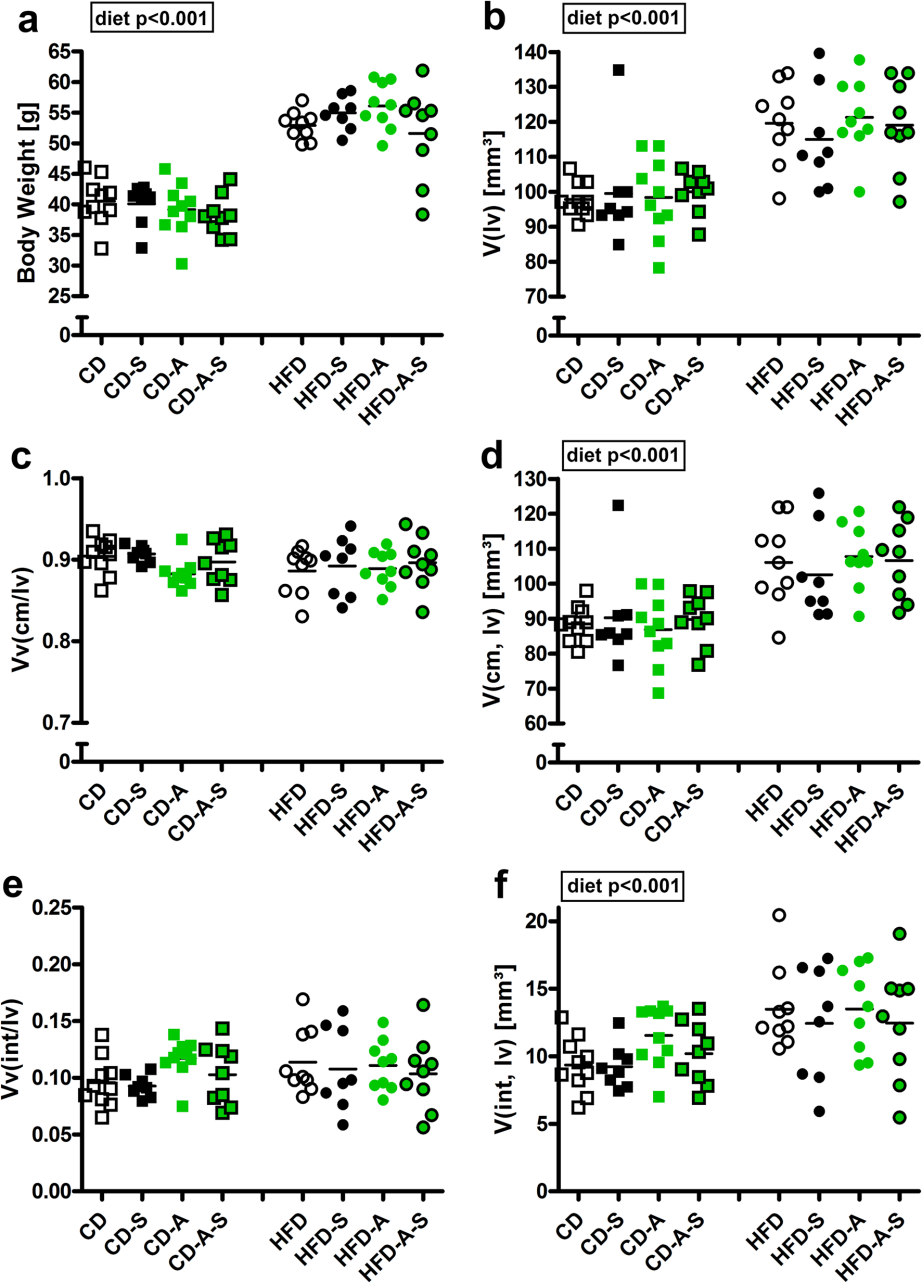
Body weights and myocardial volumes. Mice were fed control diet (CD) or high fat diet (HFD) and were left untreated, were supplemented with spermidine (-S), had access to running wheels (-A), or a combination of both (-A-S) for 30 weeks. **a** Body weights, **b** total left ventricular volume (*V*(lv)), **c** volume density of left ventricular cardiomyocytes (*Vv*(cm/lv)), **d** total volume of left ventricular cardiomyocytes (*V*(cm, lv)), **e** volume density of left ventricular interstitium (*Vv*(int/lv)), **f** total volume of left ventricular interstitium (*V*(int, lv)). Symbols represent individual animals, bars represent group means. Data were compared by 3-way ANOVA (overall significant effects are shown in boxes) and subsequent pairwise comparison by Tukey test

Control mice had a similar body weight irrespective of spermidine supplementation or voluntary activity. Mice fed high-fat diet had significantly larger body weights than control mice. No treatment effect on body weight was observed. Similarly, the volume of the left ventricle as well as the volume of left ventricular cardiomyocytes and interstitium were higher in high-fat than in control diet fed mice but no effect of the treatments was observed (Fig. 1).

**Fig. 2:**
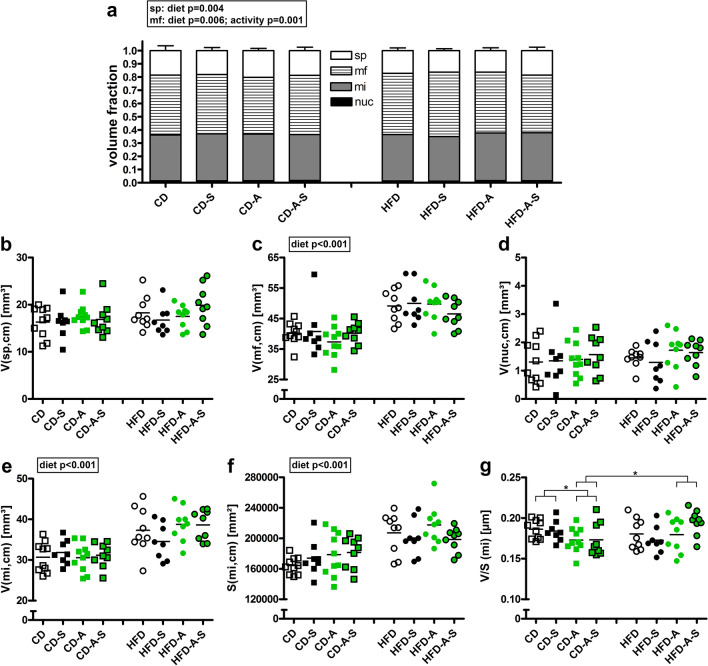
Cardiomyocyte subcellular compartments. Mice were fed control diet (CD) or high fat diet (HFD) and were left untreated, were supplemented with spermidine (-S), had access to running wheels (-A), or a combination of both (-A-S) for 30 weeks. **a** Volume densities of cellular compartments in cardiomyocytes (*sp* sarcoplasm, *mf* myofibrils, *mi* mitochondria, *nuc* nuclei), **b** total volume of sarcoplasm (*V*(sp,cm)), **c** total volume of myofibrils (*V*(mf,cm)), **d** total volume of nuclei (*V*(nuc,cm)), **e** total volume of mitochondria (*V*(mi,cm)), **f** total surface area of mitochondria (*S*(mi,cm)), **g** volume/surface ratio of mitochondria (*V*/*S* (mi)). Symbols represent individual animals, bars represent group means. Data were compared by 3-way ANOVA (overall significant effects are shown in boxes) and subsequent pairwise comparison by Tukey test (significant differences are indicated: *p ≤ 0.05)

HFD feeding induced a shift in the relative cardiomyocyte composition towards a higher myofibril fraction at the expense of the sarcoplasm fraction (Fig. 2a) suggesting an unbalanced hypertrophy of the cells. Total volumes of sarcoplasm and cardiomyocyte nuclei were unaltered between the groups (Fig. 2b, d) whereas mitochondria and particularly myofibril volumes were elevated in HFD fed mice (Fig. 2c, e) indicating that increases in myofibrils and mitochondria predominantly contributed to the HFD-induced hypertrophic remodeling of cardiomyocytes. This was not influenced by spermidine supplementation or voluntary activity. Similar to the higher volume of mitochondria, a diet effect on mitochondrial surface area was observed (Fig. 2f), however, the relationship between volume and surface area of mitochondria showed that activity reduced the volume-to-surface ratio of mitochondria in control mice, suggesting smaller mitochondria while this effect was abolished in HF diet fed mice (Fig. 2g).

**Fig. 3:**
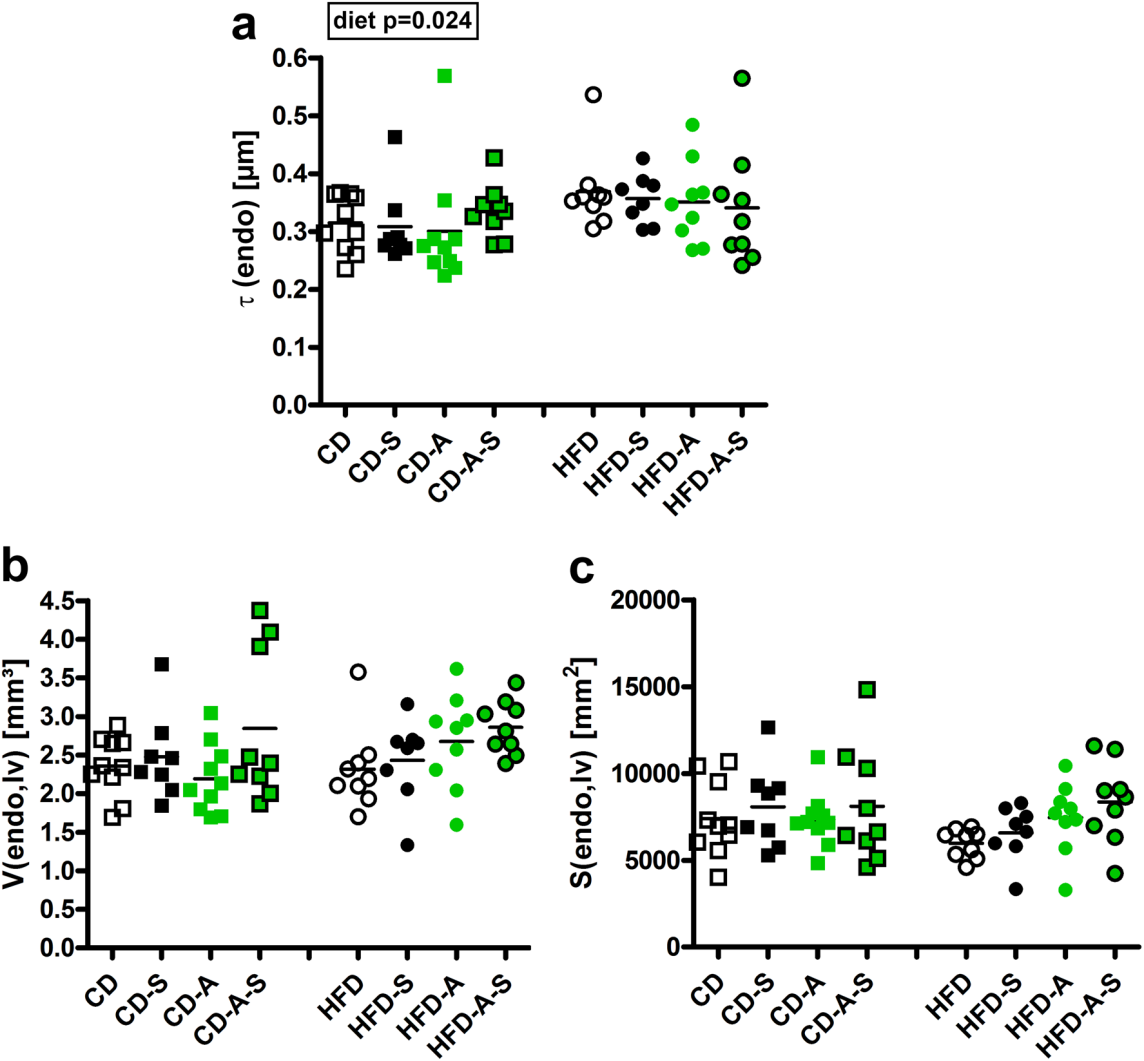
Capillary endothelial thickness, volume and surface area. Mice were fed control diet (CD) or high fat diet (HFD) and were left untreated, were supplemented with spermidine (-S), had access to running wheels (-A), or a combination of both (-A-S) for 30 weeks. **a** Thickness of endothelium (*τ*(endo)), **b** total endothelial volume (*V*(endo,lv)), **c** total endothelial surface area (*S*(endo,lv)). Symbols represent individual animals, bars represent group means. Data were compared by 3-way ANOVA (overall significant effects are shown in boxes) and subsequent pairwise comparison by Tukey test

The thickness of capillary endothelium was higher in HFD fed mice compared to controls independent of the treatments (Fig. 3a). In contrast, the surface area of the luminal endothelial surface and the volume of capillary endothelial cells did not differ between the animals (Fig. 3b, c).

**Fig. 4:**
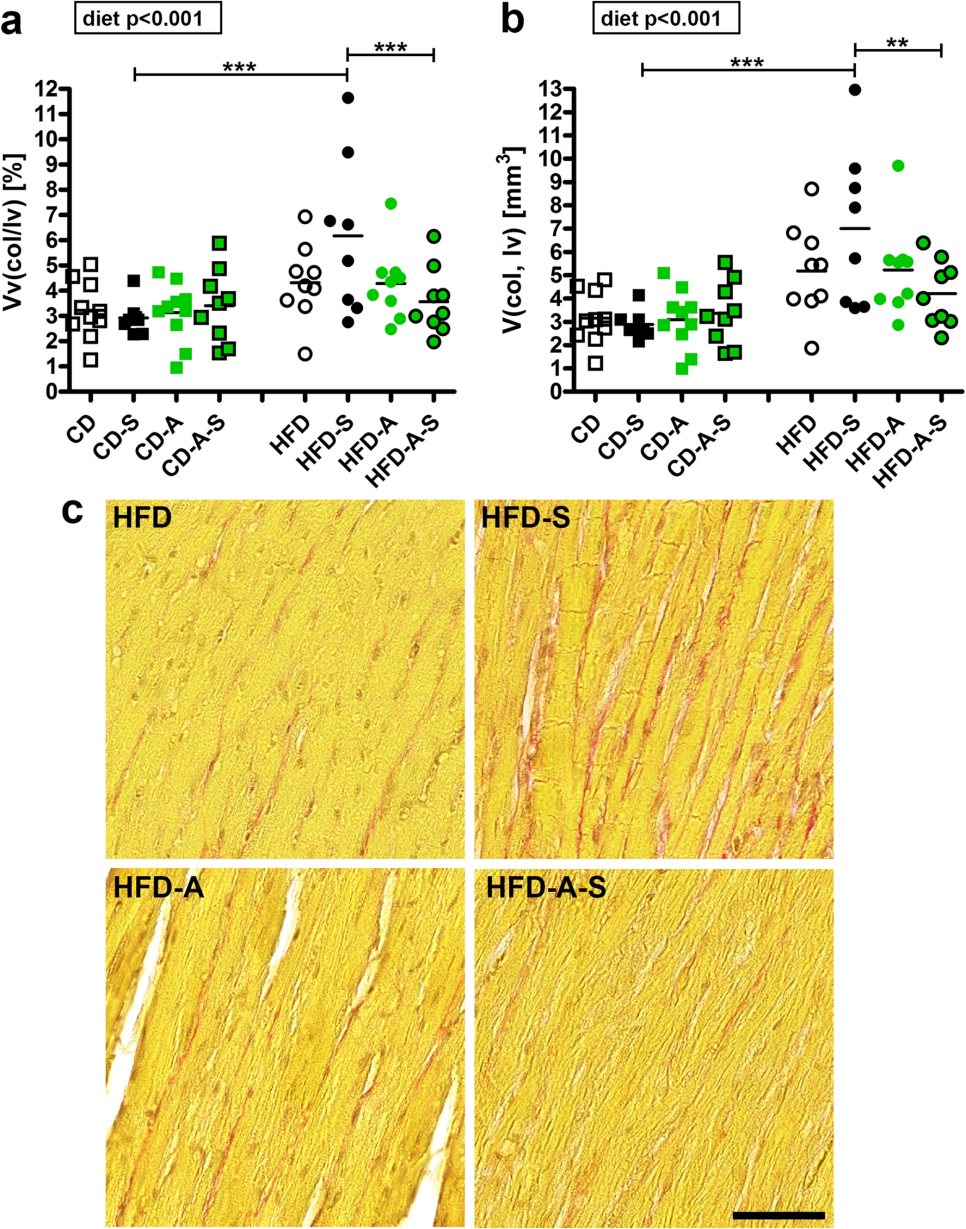
Myocardial collagen content. Mice were fed control diet (CD) or high fat diet (HFD) and were left untreated, were supplemented with spermidine (-S), had access to running wheels (-A), or a combination of both (-A-S) for 30 weeks. **a** volume density of left ventricular collagen (*Vv*(col/lv)), **b** total volume of left ventricular collagen (*V*(col,lv)). Symbols represent individual animals, bars represent group means. Data were compared by 3-way ANOVA (overall significant effects are shown in boxes) and subsequent pairwise comparison by Tukey test (significant differences are indicated: *p ≤ 0.05, **p ≤ 0.01, ***p ≤ 0.001). **c** Representative images showing collagen deposition in HFD groups, scale bar = 50 µm

Quantification of collagen fiber volume revealed a significant diet-induced increase in relative and absolute collagen fibers in all HFD groups but most pronounced in the spermidine treated HFD mice (Fig. 4). The higher relative collagen content (Fig. 4a) and the unchanged relative cardiomyocyte volume (Fig. 1c) indicates that collagen accumulation exceeds the hypertrophy of cardiomyocytes.

**Fig. 5:**
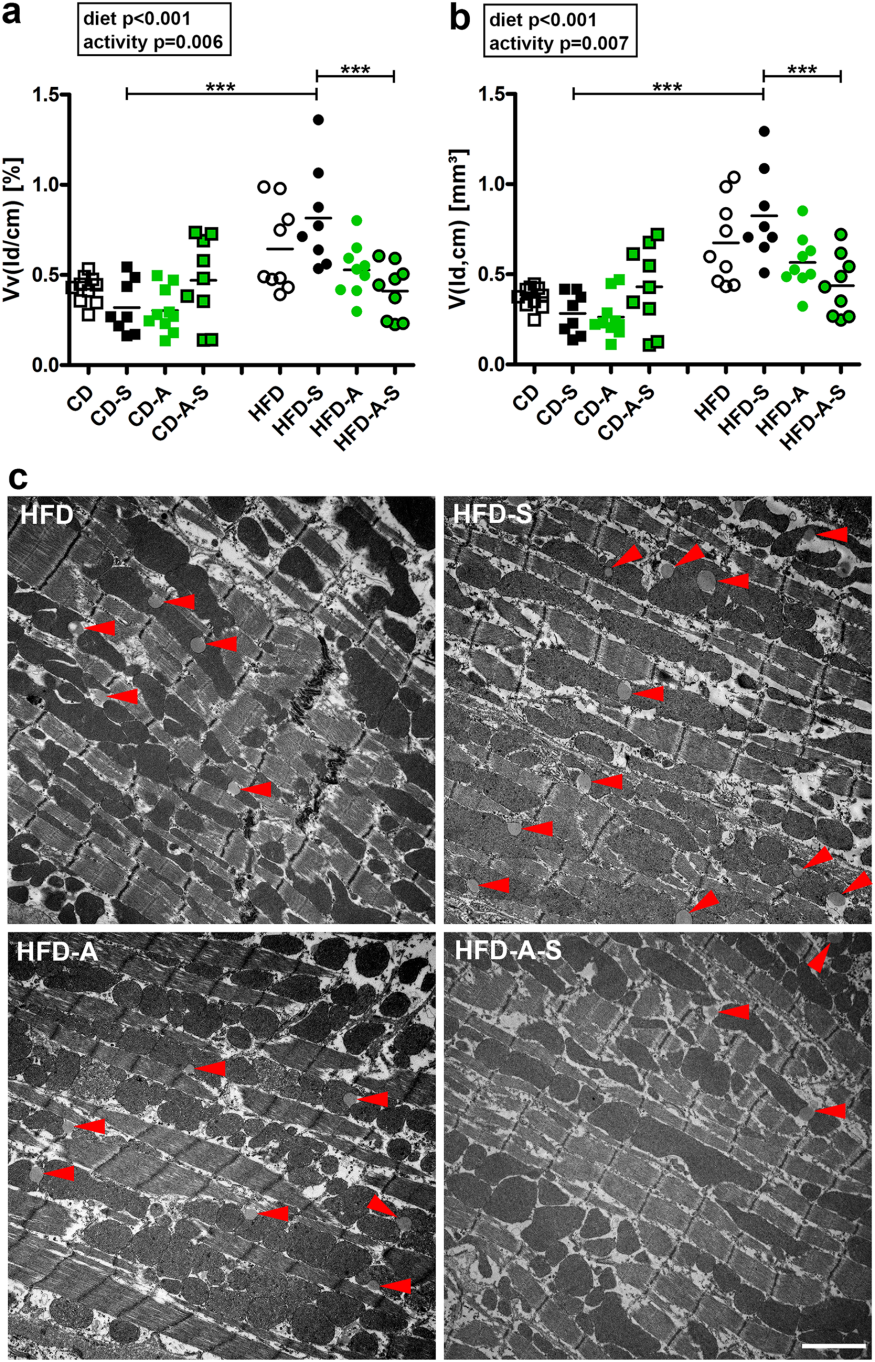
Lipid accumulation. Mice were fed control diet (CD) or high fat diet (HFD) and were left untreated, were supplemented with spermidine (-S), had access to running wheels (-A), or a combination of both (-A-S) for 30 weeks. **a** Volume densities of lipid droplets in cardiomyocytes (*Vv*(ld/cm)), **b** total volume of lipid droplets in cardiomyocytes (*V*(ld,cm)). Symbols represent individual animals, bars represent group means. Data were compared by 3-way ANOVA (overall significant effects are shown in boxes) and subsequent pairwise comparison by Tukey test (significant differences are indicated: *p ≤ 0.05, **p ≤ 0.01, ***p ≤ 0.001). **c** Representative images showing lipid accumulation in cardiomyocytes of HFD groups, scale bar = 2 µm

**Fig. 6:**
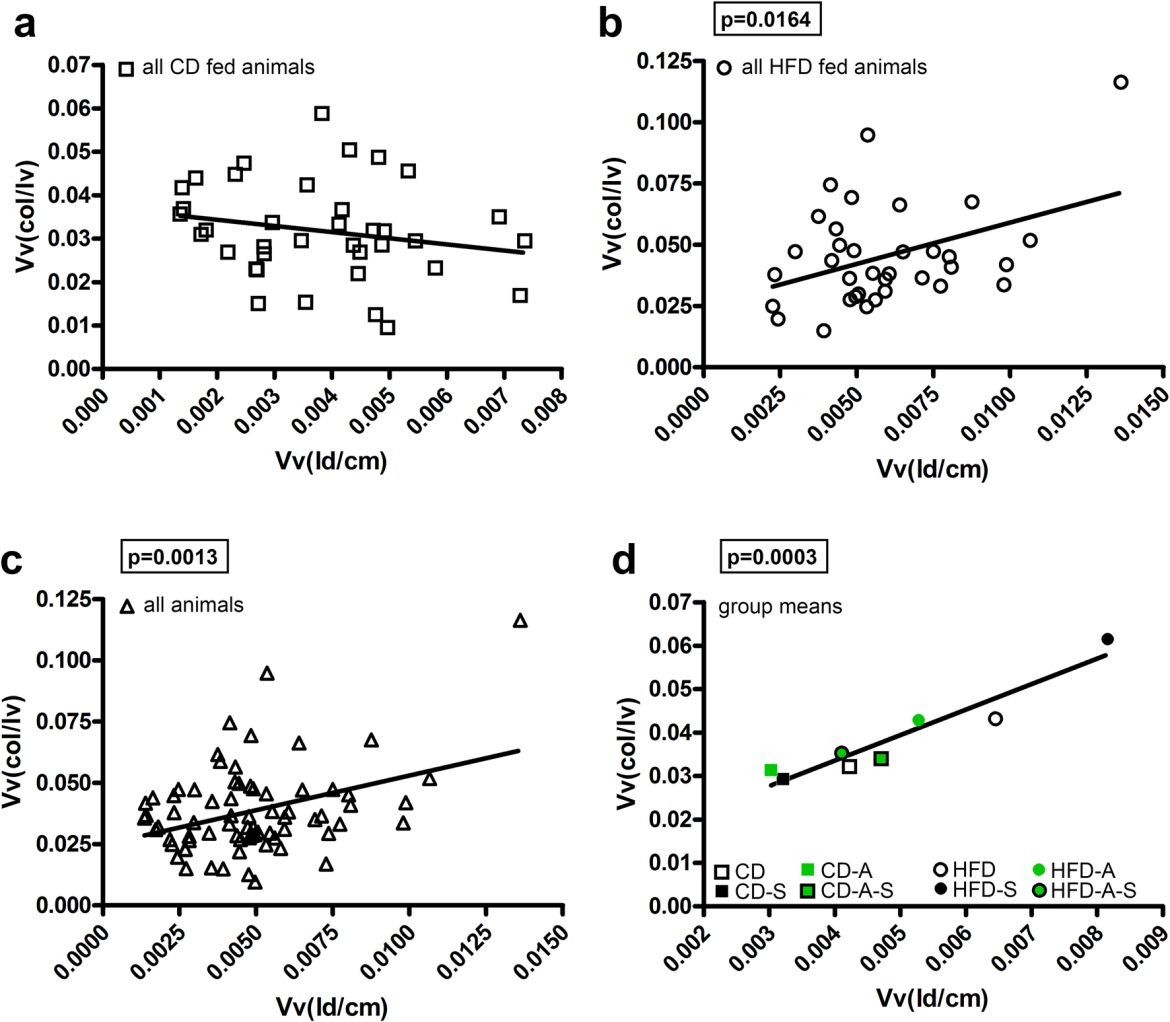
Correlation between myocardial collagen content and lipid accumulation. Plotted are volume densities of left ventricular collagen fibers (*Vv*(col/lv)) on the y axis in relation to volume densities of lipid droplet volumes in cardiomyocytes (*Vv*(ld/cm)) on the x axis. **a** Values of all CD fed mice, *b* values of all HFD fed mice, **c** values of all mice, **d** group means. **a**–**c** Symbols represent individual animals, **d** symbols represent group means. Linear regression curves are shown. Pearson correlation analysis was performed, significant *p* values are shown in boxes

As expected, cardiomyocytes of HFD fed mice contained a greater volume of lipid droplets between myofibrils and in close proximity to mitochondria with the highest values in spermidine supplemented HFD fed animals (Fig. 5). No treatment effect was observed among animals fed control diet. In contrast, activity led to smaller lipid droplet volumes with or without spermidine supplementation in mice fed HFD. This suggests that spermidine increased the lipid accumulation in cardiomyocytes whereas activity reversed the effect to a lipid volume level similar to active HFD fed mice without spermidine supplementation (Fig. 5). Interestingly, the group pattern of collagen content and lipid droplet volume was very similar. Correlation analysis showed a significant correlation between volume fraction of lipid droplets and volume fraction of collagen in mice fed high-fat diet but not in mice fed control diet. The means of all groups, however, showed a clear and highly significant correlation between these two parameters (Fig. 6) suggesting a mechanistic link between cardiomyocyte lipid accumulation and interstitial fibrosis.

## Discussion

The present study investigated the effects of a prolonged period of high fat diet with or without spermidine supplementation, voluntary activity or combination of both interventions. Overall, HFD induced cardiac hypertrophy as shown by increased volumes of left ventricle, cardiomyocytes, interstitium, myofibrils and cardiomyocyte mitochondria as well as thickening of capillary endothelium. Neither of the treatments had an effect on the hypertrophic and endothelial response. However, HFD also induced interstitial fibrosis as shown by increased collagen fiber content and cardiomyocyte lipid droplet accumulation. Both effects were most pronounced in spermidine treated animals whereas activity had a lowering effect on the spermidine-induced fibrosis and lipid accumulation. Furthermore, cardiomyocyte mitochondria had a smaller volume to surface ratio in active mice fed CD which was not the case in HFD fed mice.

The structural characteristics investigated in this study were selected for the following reasons: Interstitial and collagen volume provide a direct measure of the fibrotic state of the myocardium (Schipke et al. 2017) whereas the volume of lipid droplets allows an evaluation of the diet-induced accumulation of neutral lipids in the cardiomyocytes. The volume (density) of mitochondria has been shown to be strongly correlated with the oxidative capacity, thus providing a direct structure–function relationship which can be much more efficiently estimated than the surface area of the inner mitochondrial membrane (Schwerzmann et al. 1989). In contrast, the surface area of the outer mitochondrial membrane and particularly the ratio between volume and surface area of mitochondria provides an estimate of the mean size of mitochondria at the time of fixation (Schmiedl et al. 1990). According to the law of diffusion, another functionally relevant estimate is the surface area of capillary endothelium which provides an estimate of the oxygen diffusion area for supply of cardiomyocytes (Mühlfeld et al. 2005).

Obesity is known to be associated with left ventricular hypertrophy both in humans and laboratory animals (Lauer et al. 1991; Gruber et al. 2012; Gonçalves et al. 2016) leading to a decrease in heart-to-body weight ratio (Dong et al. 2006, 2007). Previous reports on the morphological alterations in obese mice or rats range from myocyte hypertrophy and fibrosis over lipid droplet accumulation and sarcomere disorganization to mitochondrial damage and loss of mitochondria (Christoffersen et al. 2003; Leopoldo et al. 2010; Dong et al. 2007; Lino et al. 2020). In line with previous reports from our work group in a similar obesity model (Gruber et al. 2012; Schipke et al. 2014), the present study confirmed hypertrophy, fibrosis and lipid accumulation but not sarcomere or mitochondrial damage in the HFD fed mice. Obesity-related left ventricular hypertrophy was previously described to result from larger but fewer cardiomyocytes (Schipke et al. 2014).

The accumulation of lipid in the heart is associated with oxidative stress and inflammation and is closely linked with the development of myocardial fibrosis (Schram and Sweeney 2008). The connection between lipid accumulation and fibrosis is further substantiated by studies showing that cardiac lipid accumulation potentiates the effects of angiotensin II on the heart via TGFβ signaling (Glenn et al. 2015). Importantly, obesity and myocardial lipid accumulation are associated with a dysregulation of autophagy which is supposed to shift the cellular homeostasis to apoptosis (Sun et al. 2019; Varma et al. 2018).

Voluntary activity is known to have beneficial effects on the cardiovascular system (Bronikowski et al. 2003). In a stereological analysis, 4 weeks of voluntary activity increased the total volume and number while decreasing the mean volume of mitochondria (Eisele et al. 2008). In the present study, voluntary activity did not increase the total volume of mitochondria in mice fed control diet. One of the reasons for this discrepancy could be the different duration of the experiments because the running distance presented by Eisele et al. (2008) during 4 weeks was similar to the running distances in the present experiments but declined significantly until week 30. However, in the present study voluntary activity decreased the volume to surface ratio of mitochondria, a parameter which is proportional to the diameter, indicating a similar effect on mean mitochondrial volume as observed by Eisele et al. (2008). The analysis of mitochondria by electron microscopy relies on their state at the moment of fixation despite fusion or fission events constantly present in the living state. However, it can be concluded that voluntary activity led to a higher number of smaller mitochondria in the present experiments, which is possibly explained by a shift of the mitochondrial fusion-fission relationship favoring mitochondrial fission. These changes which increase the mitochondrial surface area while keeping the total volume constant were abolished by high fat diet.

Spermidine has gained considerable interest in the past decade by promoting longevity (Eisenberg et al. 2009; Filfan et al. 2020) and because of a wide range of potential organ-related beneficial effects, including cardioprotection in aging and hypertension (Eisenberg et al. 2016), decreased development of experimentally induced aortic aneurysms (Liu et al. 2020), neuroprotection in aging-associated cognitive loss (Wirth et al. 2018), liver protection in non-alcoholic fatty liver disease (Gao et al. 2018), or kidney protection after ischemic injury (Kim 2017), to name just a few. Evidence suggests that the underlying mechanism of the spermidine effects is the induction of autophagy (Madeo et al. 2019). On the other hand, a recent study has questioned the value of in vitro studies adding spermidine to cell culture medium with bovine serum because they are potentially confounded by the interaction of bovine serum with spermidine. The study suggests that the observed autophagy induction by spermidine in vitro is rather an artifact than a true biological effect (Holbert et al. 2020). However, in vivo studies have also demonstrated elevated levels of autophagy after spermidine administration.

Leaving these discrepancies as a side note, the present study showed the strongest diet-induced increase in both lipid droplet volume and collagen content in mice fed HFD and supplemented with spermidine. These data indicate that spermidine aggravated the HFD-induced lipid accumulation in cardiomyocytes and thereby contributed to myocardial fibrosis. Importantly, spermidine had no such effect under control diet conditions. This suggests that the overproportional lipid accumulation is no direct effect of spermidine but is rather based on an interaction between the cellular disturbances induced by HFD and spermidine. For example, cell culture and mouse experiments indicate that high lipid burden leads to increased autophagosome generation but less autophagosome degradation (Zhang et al. 2019). Additional interference with the dysregulated autophagic machinery by exogenous spermidine might therefore increase the imbalance and have adverse effects. Our data appear to be in contrast to a recent study which reported an anti-fibrotic effect of spermidine in streptozotocin-induced diabetes of the rat (Hu et al. 2020). However, the heart weight reported by Hu et al. (2020) was lower in the diabetic animals providing evidence that our model differs greatly from theirs. Also, they did not present total values of collagen which would be necessary to evaluate whether a true increase in collagen has taken place.

The beneficial role of moderate exercise in cardiovascular health and disease is well documented (Sharma et al. 2015). Here, in HFD fed mice, voluntary activity abolished the negative spermidine effects on lipid droplet volume and collagen fiber content down to levels similar as the respective control diet groups. Among the many physiological effects exerted by exercise, regulation of autophagy and particularly mitophagy plays an important role. As discussed above, the dysregulation of autophagy by HFD and spermidine might therefore be restored by voluntary activity. Interestingly, this effect was also observed when voluntarily active mice were supplemented with spermidine. This is in line with a recent study from our laboratory, where it was shown that spermidine alone had no positive effect on high fat or high sucrose diet-induced body weight changes whereas the combination of both interventions had a stronger effect than either treatment alone (Schipke et al. 2019).

In summary, the present study confirmed that long term exposure to a high fat diet results in cardiac hypertrophy, myocardial fibrosis and lipid accumulation. Oral spermidine application at a dose that had previously been shown to increase life span and convey cardioprotection in the mouse (Eisenberg et al. 2016) had no beneficial effect on hypertrophy and adverse effects on lipid accumulation and fibrosis. In contrast, voluntary activity induced smaller mitochondria, less lipid droplets and less fibrosis. These data should be taken into consideration when spermidine is used in obese individuals during clinical trials.

## Data Availability

Not applicable.
